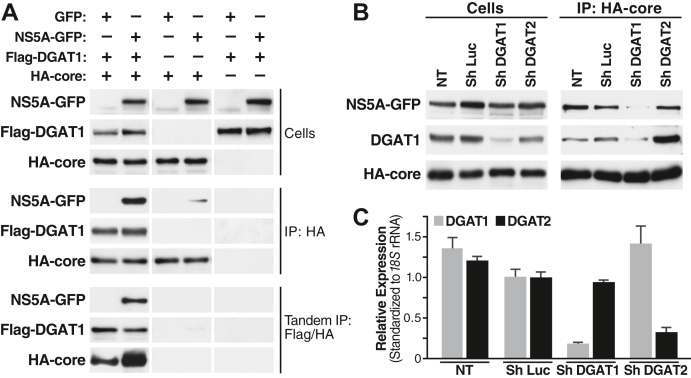# Correction: Diacylglycerol acyltransferase-1 localizes hepatitis C virus NS5A protein to lipid droplets and enhances NS5A interaction with the viral capsid core

**DOI:** 10.1016/j.jbc.2024.107975

**Published:** 2024-11-30

**Authors:** Gregory Camus, Eva Herker, Ankit A. Modi, Joel T. Haas, Holly R. Ramage, Robert V. Farese, Melanie Ott

In Figure 2A, the third column of the Flag-DGAT1 row of the Cells group of panels was erroneously duplicated as the first row of the Flag-DGAT1 row of the IP:HA group of panels. The third column of the Flag-DGAT1 row of the IP:HA group of panels was also erroneously duplicated as the third column of the HA-core row of the IP:HA group of panels. A revised version of the figure has been supplied with this correction.

**Figure:**